# Complete Duplex Right Kidney With Upper Moiety Hydronephrosis due to Ureteral Stone: A Rare Case

**DOI:** 10.1155/carm/3800537

**Published:** 2025-09-02

**Authors:** I. B. G. A. Mahesvara, I. W. Suarsana, I. B. O. W. Putra

**Affiliations:** Department of Urology, Wangaya General Hospital, Denpasar, Bali, Indonesia

**Keywords:** duplex kidney, stone formation, ureteroscopy, urinary tract symptoms

## Abstract

Duplex kidneys, though relatively uncommon (occurring in 1%–4% of the cases), can pose significant clinical challenges when associated with urinary tract symptoms. Complications requiring intervention include stone formation, urinary obstruction, and reflux disease. We present the case of a 22-year-old male with complete duplex right kidney with upper moiety hydronephrosis due to a distal ureteral stone, diagnosed by ultrasonography and confirmed with intraoperative retrograde pyelography (RPG). Definitive management was achieved via ureteroscopy (URS) lithotripsy.

## 1. Introduction

Duplex kidney is a rare developmental anomaly affecting approximately 1% of healthy adults. While often asymptomatic, it becomes clinically significant when associated with urinary tract symptoms, which occur in 1%–4% of the cases. Due to its frequently silent presentation, the true incidence of this malformation is likely underreported. In adults, duplex kidneys are typically present as an incidental finding, a nonfunctional moiety, or calculus disease [1].

Duplication results from the formation of two independent ureteric buds originating from the same Wolffian duct. Its anatomical variations depend on the extent of fusion, ranging from a bifurcated renal pelvis or partially duplicated ureter to incomplete duplication (where ureters merge close to or within the bladder) or complete duplication (with distinct ureteral openings into the bladder) [2]. The term “duplex kidney” refers to a renal unit with dual pelvicalyceal systems, accompanied by separate renal vasculature, collecting systems, and ureters. The embryogenesis of the urinary tract is a highly regulated process involving not only renal development but also morphogenesis of the lower urinary tract [3]. The clinical significance of these anomalies depends on ureteric bud positioning, metanephric interaction, and associated malformations. The clinical presentation varies based on ureteral orifice location, patient sex, and concurrent abnormalities [4].

This report describes a case of an adult with a duplex kidney and ureteral stone. This patient remained asymptomatic until he developed symptoms of abdominal pain in the right lumbar and inguinal regions in adulthood.

## 2. Case Report

A 22-year-old male patient presented to our urology department due to dull abdominal pain in right lumbar region, occasionally radiating to the inguinal region. He reported that the pain had been intermittent for 9 months and progressively worsened in 3 days prior to his admission. On daily basis, he was continent. Two months earlier, the patient had sought treatment at another hospital for the same complaint. At that time, he underwent right ureteroscopy (URS) due to suspected right ureteral stone and hydronephrosis. However, the treating physician found no stone during the procedure, suggesting it may have passed spontaneously or retropulsed and DJ stent was subsequently placed.

**Figure 1:**
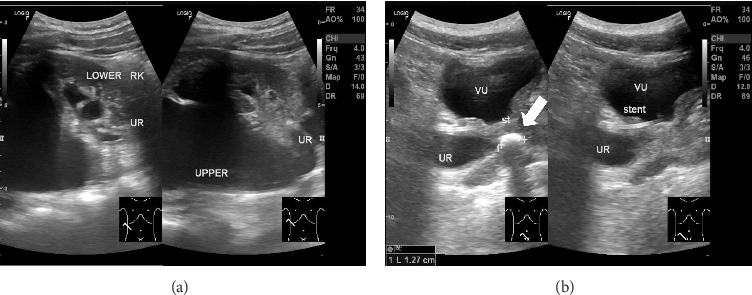
(a) Right kidney ultrasound showing: grade III hydronephrosis (upper moiety), grade I hydronephrosis (lower moiety), and grade I dilation of the lower moiety pelvicalyceal system (with ureteral stent in situ). (b) Right distal ureteric stone was observed originating from the upper moiety and ureteral stent was placed in the lower moiety.

Tenderness was noted over the right flank during percussion. Complete blood count (CBC) was within normal limits (WBC: 8.22, HGB: 15.3, HCT: 46.2, and PLT: 241). Ultrasonographic findings were inconclusive regarding a right renal duplex system. However, hydronephrosis was definitively identified with grade III hydronephrosis of the upper moiety, grade I hydronephrosis of the lower moiety (with a stent in situ), and a distal ureteric stone measuring approximately 12.7 mm in size (Figure 1).

**Figure 2:**
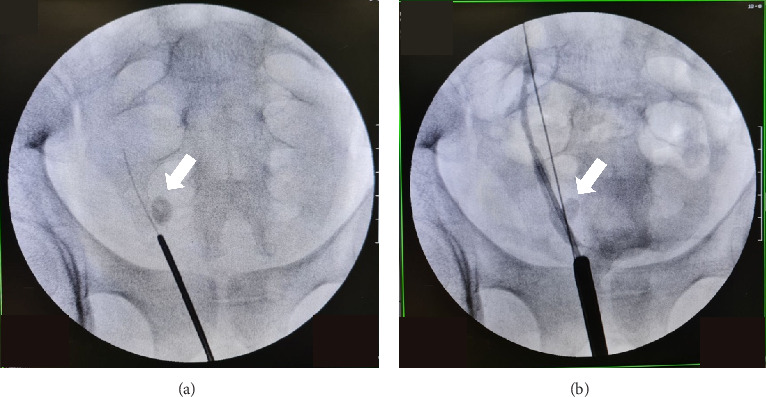
(a) Right distal ureteric stone seen on plain fluoroscopy. (b) Right RPG revealed the stone located at a different tract (not on the native ureter).

**Figure 3:**
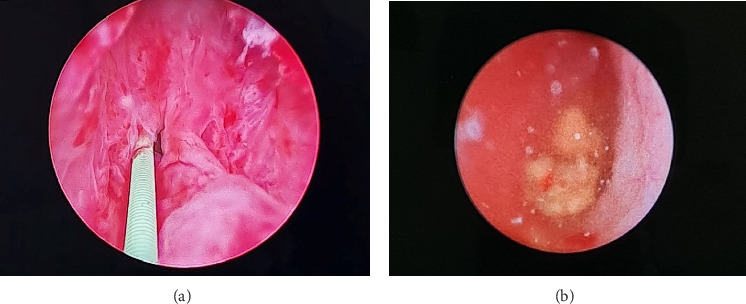
(a) Ectopic orifice located on prostatic urethra with wire. (b) Ureteral stone identified at an ectopic ureter which drained within the prostatic urethra, positioned at the right paraverumontanum area.

Rigid URS was performed using a 6/7.5Fr URS. Both native ureteral orifices were identified at their respective locations on the lateral bladder trigone. Under fluoroscopy guidance, a retrograde contrast study was performed but the stone appeared to be located in a different ureter (Figure 2). Upon further exploration, an ectopic ureteral orifice was found at the right paraverumontanum area. URS was then performed under guidewire and fluoroscopy guidance (Figure 3). The ureteral stone was identified, fragmented, and evacuated. Retrograde pyelography (RPG) revealed that the ureter containing the stone drained a dilated upper moiety of the right kidney. A ureter catheter was inserted at the end of the procedure to ensure adequate drainage of the upper moiety. The catheter was removed on postoperative day two, and the patient was discharged without complaints. A 3-month postdischarge follow-up in our outpatient clinic was advised, but the patient did not return for further evaluation.

## 3. Discussion

The term duplex kidney refers to renal unit with two pelvicalyceal systems. A complete duplex kidney is said to be a two distinct pelvicalyceal systems that developed from two ureteral buds from the mesonephric duct; as a result, this produced two ureters. Depending on the renal segment that they drain, ureters are classified as upper or lower moiety [1]. In complete duplication, the upper pole moiety is more frequently affected by abnormalities than the lower pole moiety. Hydronephrosis is commonly observed, usually caused by an obstructive process [5].

At present, ectopic ureter is more frequently seen in duplicated systems than in single systems. A study mentioned that females experience this condition two to four times more frequent than males. A duplex kidney is usually asymptomatic and incidental, often without clinical significance. However, complications may arise depending on the affected moiety. The upper moiety can involve ectopic ureteric insertion (with or without ureterocele) or multicystic dysplasia, while the lower moiety may develop vesicoureteral reflux (VUR), renal scarring, or ureteropelvic junction (UPJ) obstruction. Though often benign, these risks warrant careful evaluation when detected [6]. Urinary incontinence is the most common clinical manifestation in females. While in males, they frequently complain of flank pain, fever, UTI, abdominal masses, or epididymitis in this disease. Prostatic urethra (57%) is the most prevalent outflow for ectopic ureter in males, followed by seminal vesicles (33%), the ejaculatory duct (10%), and the vas (10%) [3]. Our case had a complete duplex right kidney with upper moiety hydronephrosis secondary to distal ureteral calculi which drained into the prostatic urethra just above the external sphincter. The patient was continent but symptomatic due to the ureteral obstruction. Urinary stones can form in duplex kidney patients as a complication [7].

Early diagnosis with proper imaging studies and prompt treatment will reduce complication and deterioration of renal function. Contrast study prior to intervention such as CT urography is beneficial for accurate diagnosis [8]. In our case, the patient declined the CT scan due to concerns regarding cumulative radiation exposure. Ultrasound examination revealed a suspected duplex right kidney with a distal ureteric stone. During URS, we performed RPG and identified that the stone was located not on the native ureter. As described by the Weigert–Meyer law, the consistent anatomical pattern in duplex kidneys is critical for understanding related pathologies. It states that the ureter from the upper renal pole often has an ectopic, obstructed connection, while the ureter from the lower pole is prone to reflux. It explains how the ureters from the upper and lower renal moieties insert into the bladder and the associated complications. We searched for a possible more distal ureteric orifice and identified an opening lateral right to the verumontanum on the prostatic urethra. Based on our experience, we do recommend a contrast study prior to surgical intervention for a more safe and accurate surgical procedure.

## 4. Conclusion

This case demonstrates the diagnostic and therapeutic challenges of complete duplex kidney systems with ectopic ureteral insertion. The patient presented with obstructive symptoms due to a distal ureteral stone in an ectopic upper moiety ureter draining into the prostatic urethra, a finding consistent with the Weigert–Meyer law. While ultrasonography suggested the diagnosis, intraoperative RPG was crucial for definitive anatomical mapping and successful stone management via URS. We emphasize that comprehensive imaging evaluation prior to surgical intervention optimizes outcomes in these complex anatomical variants.

## Data Availability

Data sharing is not applicable to this article as no datasets were generated or analyzed during the current study.
